# True management of Obstructed Hemi-vagina and Ipsilateral Renal Anomaly syndrome

**DOI:** 10.4274/tjod.23434

**Published:** 2016-12-15

**Authors:** Betül Yakıştıran, Yavuz Emre Şükür, Batuhan Turgay, Cem Atabekoğlu

**Affiliations:** 1 Ankara University Faculty of Medicine, Department of Obstetrics and Gynecology, Ankara, Turkey

**Keywords:** Hematometrocolpos, mullerian duct anomaly, obstructed hemi-vagina and ipsilateral renal anomaly syndrome, uterus didelphys

## Abstract

Herlyn-Werner-Wunderlich syndrome is an unusual congenital anomaly of the female genitourinary system, which is described as uterine didelphys with Obstructed Hemi-vagina and Ipsilateral Renal Anomaly (OHIRA), also known as OHVIRA syndrome. Typical symptoms are pelvic pain, tenderness, pelvic mass due to blood collection in the obstructed hemi-vagina and uterus, and dysmenorrhea that usually begins shortly after menarche. Clinical suspicion is very important for diagnosis and correct management avoids both short- and long-term complications. Surgical removal of the vaginal septum is the main treatment method. Herein, we describe the evaluation and surgical management of a patient with OHVIRA syndrome who was diagnosed using magnetic resonance imaging and pelvic ultrasound.

## PRECIS:

The management of a girl aged 13 years with obstructed hemivagina and ipsilateral renal anomaly syndrome is presented.

## INTRODUCTION

Obstructed hemi-vagina and ipsilateral renal anomaly (OHVIRA) syndrome, traditionally known as Herlyn-Werner-Wunderlich syndrome, is a rare clinical entity of Müllerian anomalies, which has been reported as case reports since 1922. Obstructive mullerian anomalies are estimated to affect approximately 0.1-3.8% of the female population^(1)^. It mainly presents with cyclical and or chronic pelvic pain and pelvic swelling while having regular cycles due to hematometrocolpos in the obstructed hemi-vagina. The classic presentation of OHVIRA syndrome is that of a postmenarchal girl with remittent pelvic pain, usually associated with menstruation, and a vaginal bulge on pelvic examination and/or foul discharge. The growing experience of OHVIRA syndrome in literature has familiarized physicians with this condition. However, delays in diagnosis are still a concern that may lead to complications such as chronic infection, endometriosis, and adhesions, which result in subfertility or infertility. Herein, we describe the evaluation and surgical management of a girl with OHVIRA syndrome who was diagnosed using magnetic resonance imaging (MRI) and pelvic ultrasound examination.

## CASE REPORT

**Figure 1:**
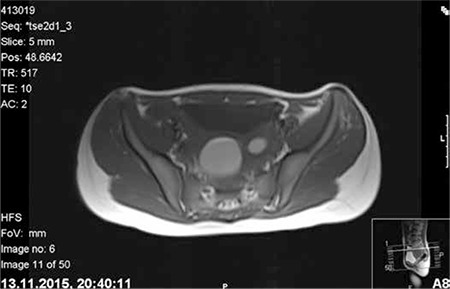
Magnetic resonance image demonstrating uterus didelphys with left hematometrocolpos

**Figure 2:**
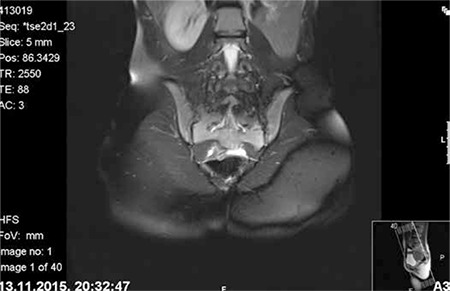
Magnetic resonance image demonstrating left renal agenesis

**Figure 3:**
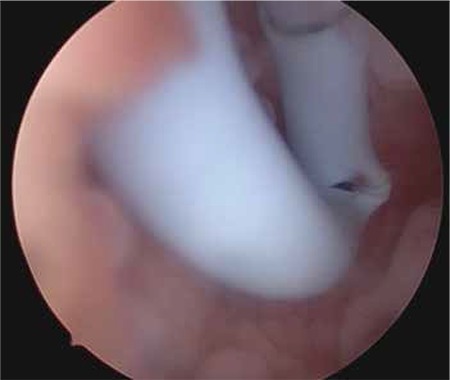
Suprapubic drainage catheter placed in the left obstructed hemi-vagina

**Figure 4:**
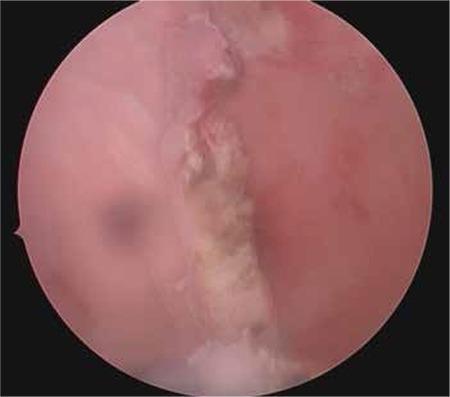
Vaginal septum and both vaginal spaces after dissection with a unipolar needle electrode

A girl aged 13 years was referred to our hospital because of cyclical pain in the lower abdomen, which she had had for the last two months, hindering her daily activities. The patient denied having any recent abdominal trauma, vomiting or diarrhea, and menarche had occurred at age 12 years. She was not sexually active and under any medical treatment. The laboratory tests including complete blood count, tumor markers, and beta human chorionic gonadotropin were within normal ranges. An abdominal ultrasound examination revealed absence of the left kidney and a cystic mass adjacent to the uterus that filled the left half of the pelvis. The uterus was of normal size and shape with myometrium, cervix, and vagina. The abdominopelvic MRI scan demonstrated an enlarged mass consistent with hematometrocolpos, a uterus didelphys, along with left renal agenesis (Figures 1, 2). In the hospital where she was first admitted, a suprapubic catheter was placed into the left obstructed hemi-vagina under ultrasound guidance by the department of interventional radiology. Although the hematometrocolpos was drained, it was not a definitive treatment. On admission to our clinic, she had no pain but a suprapubic catheter that was sutured to skin. The external genitalia were normal. After the patient and her family were informed, vaginal examination was performed under general anesthesia. Vaginoscopy and hysteroscopy were performed in the lithotomy position without hymenotomy. The left hemi-vagina was blunt. After clear visualization of the right vagina, the vaginal septum was dissected using a hysteroscopic unipolar needle electrode. The left hemi-vaginal and uterine cavities were observed, along with the suprapubic drainage catheter (Figure 3). The supra-pubic catheter placed in the left obstructed hemi-vagina was gently removed. The vaginal septum was then dissected longitudinally using a unipolar needle electrode. After dissection, a 3-cm gap in the vaginal septum was obtained, which allowed drainage from the left uterine cavity (Figure 4). The patient tolerated the procedure well and was discharged from hospital the next day. The first follow-up visit was four weeks after the operation. She had no symptoms and no complications were observed. At the sixth month follow-up visit she reported regular and painless menstrual cycles. On ultrasound examination, no distension was observed in the uterine horns or vagina.

## DISCUSSION

The Müllerian duct develops craino-caudally and fuses between the 6^th^ and 22^nd^ gestational weeks. The prevalence of Müllerian anomalies is an issue that remains to be clarified. Discrepancies of diagnostic modalities, classification systems, terminologies, and population characteristics amongst available studies rendered this issue confounding. The estimated prevalence ranges from 0.1% to 3.8%^(1)^. According to a largely accepted hypothesis, OHVIRA syndrome is caused by an embryonic arrest at about the 8th gestational week^(2,3)^. It seems that an injury on the caudal portion of the mesonephric (Wolffian) duct subsequently leads to malformation and malfusion of Müllerian ducts. Injury on the mesonephric duct also results with renal anomalies. However, there are some reported cases with obstructed hemi-vagina, double uterus, and normal urinary systems, which seem to conflict with this hypothesis. Patients present with pelvic pain, dysmenorrhea, pelvic mass, and rarely with complications such as pyocolpos, endometriosis, and infertility^(4)^. Most patients report a history of regular menses until the obstruction of the hemi-vagina resulted in distention and enlarged mass. Hence, physicians should suspect OHVIRA syndrome in young patients who present with these symptoms. Obstructed hemi-vagina and renal anomalies in OHVIRA syndrome are seen twice as frequently in the right side of the body compared with the left^(5)^. The estimated mean age of diagnosis was defined as 14 years in the literature^(2)^. This case had an unusual presentation considering the lateralization. These kinds of cases require a high amount of suspicion for diagnosis. Vaginal tissues are quite tense and the obstructed hemi-vagina may contain large amounts of blood. A sufficient enough absorption of blood between periods may prevent aggravation of the symptoms. Inability to perform vaginal examination on a virgin patient, lower accuracy of abdominal ultrasound, mild nature of symptoms, and lacking adequate amount of suspicion or experience may all contribute to delayed diagnosis and improper treatment, which may result in intra-abdominal infection and/or abscess. Early recognition is important in avoid complications.

In cases of OHVIRA, the vaginal septum is generally longitudinal and has variable thickness. Abdominal ultrasonography has been the preferred initial imaging modality; MRI should be considered for diagnosis and decision making if there is a suspicious morphology of the uterus and adnexa^(2,3)^. The association between OHVIRA syndrome and other urogenital abnormalities can be better evaluated using MRI. MRI is far better than ultrasound for characterizing anatomic relationships owing to its multiplanar capabilities and larger field of view. However, the gold standard for diagnosis is laparoscopy, which has the added benefit of performing therapeutic drainage of hematometra/hematocolpos, vaginal septotomy, and marsupialization. Treatment usually involves surgery in the form of excision of the vaginal septum, which helps to relieve the obstruction. Previously, a two-step surgical approach including drainage and resection of the septum was performed. Also, hymenotomy was favored for better visualization. However, with improved surgical capabilities, it is possible to complete the surgery in one procedure without hymenotomy. Prognosis is good, with the major concern being preservation of fertility. However, symptom recurrence due to vaginal adhesions is possible. In such a condition, silicon dilators can be used following re-resection^(6)^. Women with uterus didelphys have a high likelihood of becoming pregnant, with approximately 80% of patients able to conceive, but with elevated rates of premature delivery (22%) and abortion (74%); cesarean section is necessary in over 80% of patients^(7)^. Understanding the imaging findings is critical for early diagnosis in an attempt to prevent complications such as endometriosis or adhesions from chronic infections with subsequent infertility.

## CONCLUSION

OHVIRA syndrome is a rare congenital anomaly with different clinical presentations. Ultrasound and MRI are the initial imaging modalities and laparoscopy is the gold standard for diagnosis. The main treatment modality is the resection of the vaginal septum through vaginoscopy without hymenotomy.
